# Polyglutamine length-dependent toxicity from α1ACT in *Drosophila* models of spinocerebellar ataxia type 6

**DOI:** 10.1242/bio.021667

**Published:** 2016-12-09

**Authors:** Wei-Ling Tsou, Sultan H. Qiblawi, Ryan R. Hosking, Christopher M. Gomez, Sokol V. Todi

**Affiliations:** 1 Department of Pharmacology, Wayne State University, Detroit, MI 48201, USA; 2 Department of Neurology, University of Chicago, Chicago, IL 60637, USA; 3 Department of Neurology, Wayne State University, Detroit, MI 48201, USA

**Keywords:** Ataxia, *CACNA1A*, *Drosophila*, Gal4-UAS, Neurodegeneration, Polyglutamine, Spinocerebellar ataxia type 6 (SCA6)

## Abstract

Spinocerebellar ataxia type 6 (SCA6) is a neurodegenerative disease that results from abnormal expansion of a polyglutamine (polyQ) repeat. SCA6 is caused by CAG triplet repeat expansion in the gene *CACNA1A*, resulting in a polyQ tract of 19-33 in patients. *CACNA1A*, a bicistronic gene, encodes the α1A calcium channel subunit and the transcription factor, α1ACT. PolyQ expansion in α1ACT causes degeneration in mice. We recently described the first *Drosophila* models of SCA6 that express α1ACT with a normal (11Q) or hyper-expanded (70Q) polyQ. Here, we report additional α1ACT transgenic flies, which express full-length α1ACT with a 33Q repeat. We show that α1ACT33Q is toxic in *Drosophila*, but less so than the 70Q version. When expressed everywhere, α1ACT33Q-expressing adults die earlier than flies expressing the normal allele. α1ACT33Q causes retinal degeneration and leads to aggregated species in an age-dependent manner, but at a slower pace than the 70Q counterpart. According to western blots, α1ACT33Q localizes less readily in the nucleus than α1ACT70Q, providing clues into the importance of polyQ tract length on α1ACT localization and its site of toxicity. We expect that these new lines will be highly valuable for future work on SCA6.

## Abstract (precis)

**Summary:** The spinocerebellar ataxia type 6 (SCA6) protein, α1ACT, causes polyglutamine length-dependent toxicity and aggregation in new *Drosophila* transgenic lines, opening the door for additional studies of this disease.

## INTRODUCTION

Spinocerebellar ataxia type 6 (SCA6) is an age-dependent neurodegenerative disease caused by anomalous expansion in the CAG triplet repeat in the gene *CACNA1A* (Orr, 2012; Todi et al., 2007). Symptoms for SCA6 consist predominantly of a ‘pure’ gait ataxia that can be accompanied by tremors, visual symptoms and episodic vertigo. The CAG repeat in *CACNA1A* encodes a polyglutamine (polyQ) repeat, thus classifying SCA6 as one of the members of the polyQ family of neurodegenerative diseases. Similar to those disorders, which include Huntington's disease and several other SCAs, there is no cure for SCA6 (Du et al., 2013; Miyazaki et al., 2016; Williams and Paulson, 2008).

**Fig. 1.**
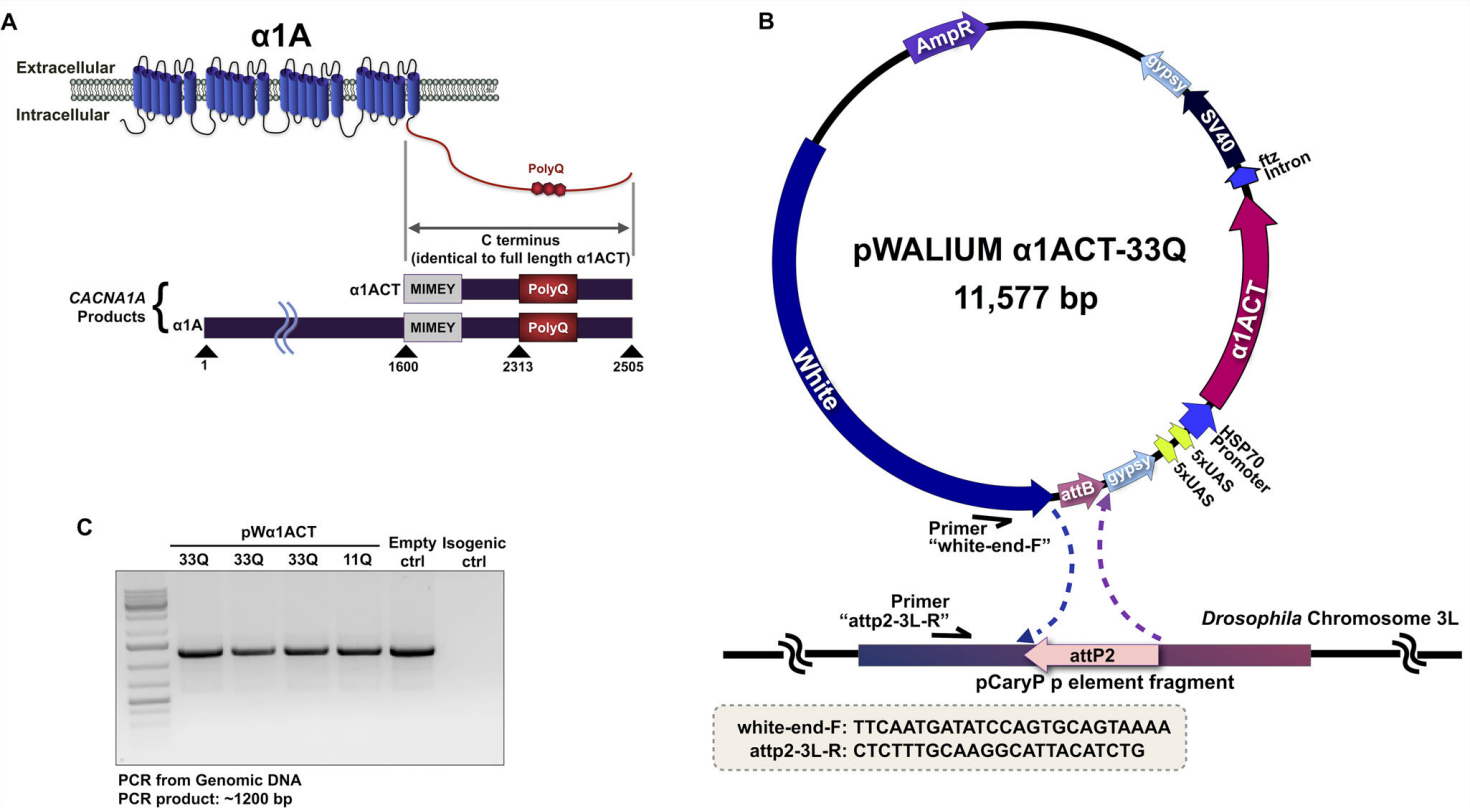
**Cloning strategy.** (A) Diagrammatic representation of the products of *CACNA1A*. (B) Cloning of α1ACT cDNA into pWALIUM10-moe and the primers used for PCRs in panel C. This version of α1ACT does not have any exogenous tags added. (C) PCR reactions from genomic DNA indicating that pWALIUM was integrated into the correct site (attP2) and in the correct orientation. Empty ctrl: pWALIUM without α1ACT. Isogenic ctrl: host line without any insertions into attP2. For 33Q, results from three independent lines are shown. Table 1 details the genotypes of flies in all figure panels.

*CACNA1A* is a bicistronic gene that encodes two proteins, the calcium channel subunit α1A and the transcription factor α1ACT, which is produced via an internal ribosomal entry site (Fig. 1A) (Du et al., 2013). Abnormal polyQ expansion in α1ACT is toxic in mice and causes a SCA6-like phenotype (Du et al., 2013; Miyazaki et al., 2016). We recently generated and characterized the first *Drosophila melanogaster* models of SCA6 which express the full-length, untagged, human α1ACT protein with a normal (11Q) or hyper-expanded (70Q) repeat (Tsou et al., 2015a). The hyper-expanded protein was highly toxic in all tissues tested in the fruit fly (Tsou et al., 2015a). Because a 70Q repeat is outside of the range observed in patients (19Q-33Q is the demonstrated pathological range), we generated flies that express α1ACT with a 33Q repeat, which we describe in this report.

The goal of the *Drosophila* models of SCA6 is to enable rapid investigations toward understanding the biology of disease in this disorder and to find therapeutic options for this type of ataxia. Here, we present evidence that the 33Q variant of α1ACT is toxic in flies, but substantially less so than the 70Q, hyper-expanded α1ACT protein. According to our genetic, structural and biochemical experiments, there is a clear relation between polyQ length and extent of toxicity from α1ACT. Additionally, the hyper-expanded version is more prominently localized to the nucleus than 33Q or 11Q variants. Together, these models should help move forward the field of SCA6 studies by offering varying degrees of toxicity, timeline of degeneration and propensity to localize in different subcellular locations.

## RESULTS

### The 33Q model of SCA6

To generate new lines of *Drosophila* that express human α1ACT (Fig. 1A) with 33Q through the Gal4-UAS system, we utilized the same protocol employed to generate α1ACT11Q and α1ACT70Q flies (Tsou et al., 2015a). The full-length α1ACT cDNA with a 33 CAG repeat was sub-cloned into the pWALIUM10.moe vector (Fig. 1B), which led to single-copy, phiC31-dependent insertion (Groth et al., 2004) into the attp2 site on *Drosophila*'s third chromosome. This is the same site of integration as α1ACT11Q and α1ACT70Q and in the same orientation (Fig. 1C; Table 1 lists the genotypes of flies in each figure) (Tsou et al., 2015a). We selected this particular expression system and vector as it includes high flexibility to control the extent to which the toxic protein is expressed (Markstein et al., 2008; Ni et al., 2008). Five of the ten upstream activating sequences (UAS) in pWALIUM are bordered by loxP sites that enable investigators to have as few as five or as many as twenty UAS in a single fly, thus providing great titrating power for protein levels and the phenotypes they cause.

**Table 1.**
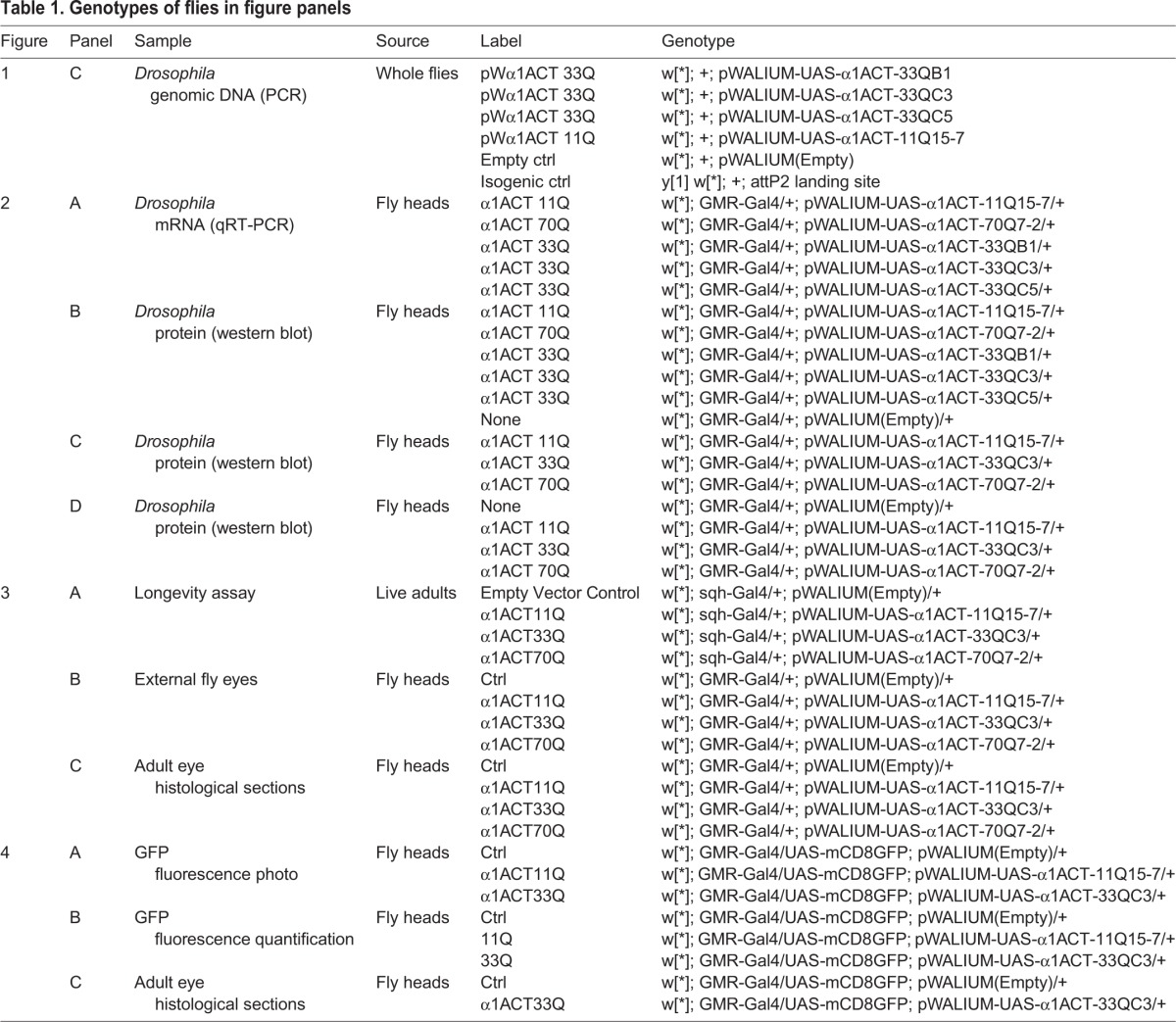
**Genotypes of flies in figure panels**

**Fig. 2.**
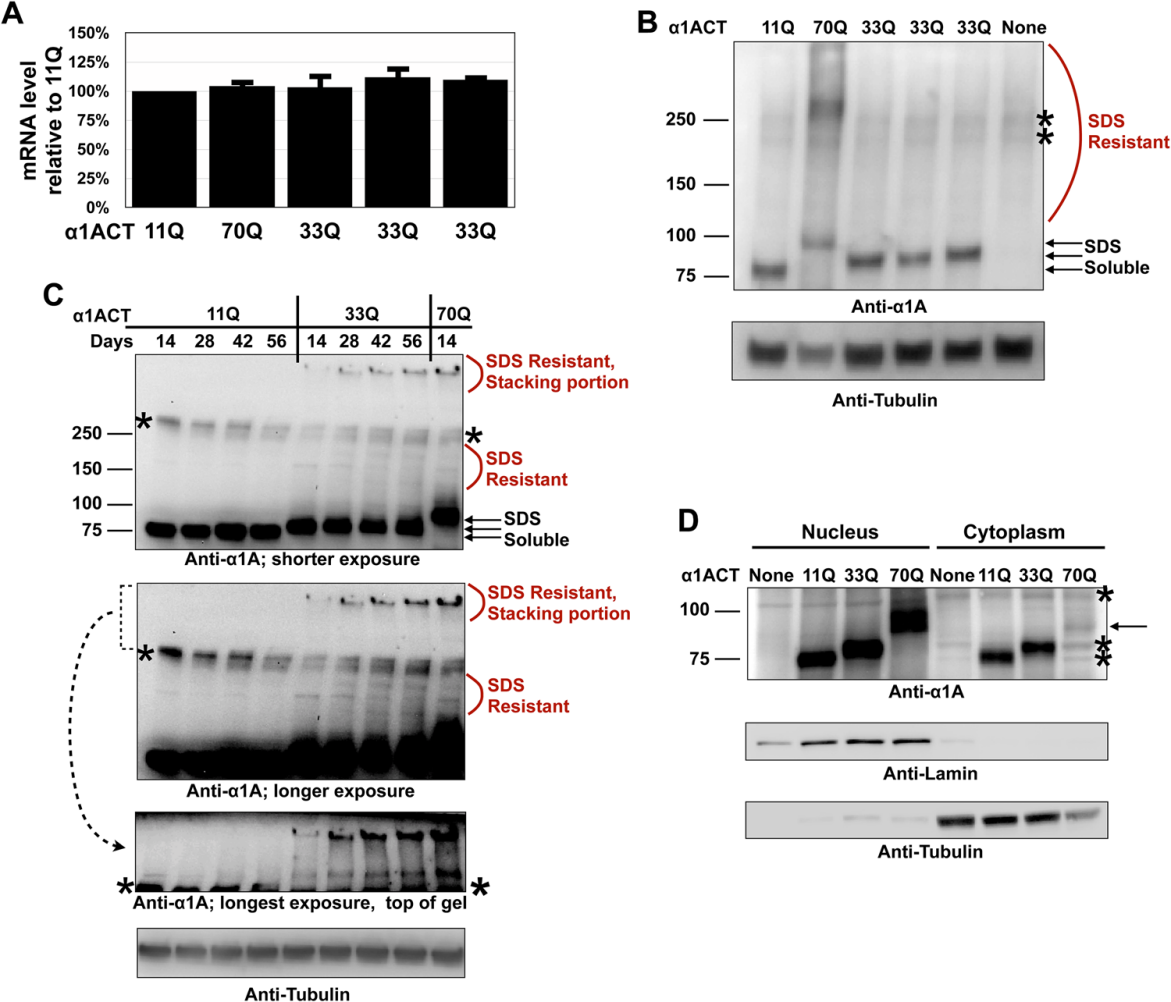
**Expression of α1ACT in flies.** (A) qRT-PCR reactions with the indicated α1ACT polyQ lengths expressed in fly eyes through the GMR-Gal4 driver. Ten fly heads per reaction were used. *N*=3 independent experiments. Error bars indicate s.d. Flies were heterozygous for driver and α1ACT transgenes. For 33Q, results from three independent lines are shown. (B) Western blots from flies with genotypes as in panel A. Fifteen fly heads per group were used. Asterisks indicate non-specific bands. ‘None’ denotes pWALIUM without α1ACT in the presence of GMR-Gal4; SDS-resistant label pertains only to the 70Q variant of α1ACT. Arrows indicate SDS-soluble species of 11Q, 33Q and 70Q α1ACT. Flies were one day old. (C) Western blots from flies with genotypes as in panels A and B, aged for the indicated amount of time. Fifteen fly heads per group were used. SDS-resistant label pertains to 33Q and 70Q α1ACT. We do not notice similar species with the 11Q variant. Asterisks indicate non-specific bands. Arrows indicate SDS-soluble species of 11Q, 33Q and 70Q α1ACT. (D) Subcellular fractionation of dissected fly heads with genotypes as in panel A. Asterisks indicate non-specific bands. Arrow indicates faint signal from α1ACT70Q present in the cytoplasmic fraction.

As shown in Fig. 2A and B, when driven by GMR-Gal4 selectively in fly eyes, UAS-dependent expression of the three different versions of α1ACT leads to comparable mRNA and protein levels. Unlike the other two variants, the 70Q, hyper-expanded α1ACT protein shows both SDS-soluble and SDS-resistant species in one-day-old flies (Fig. 2B). Over time, the 33Q variant also shows the presence of SDS-resistant species (Fig. 2C). In 56-day-old adults, SDS-resistant species in 33Q-expressing eyes are comparable to those in 14-day-old, 70Q-expressing eyes. This is especially noticeable at the top of the membrane (Fig. 2C, lower panels). We do not observe similar species with 11Q α1ACT over this same time course (Fig. 2C and other supportive data not shown).

α1ACT is a transcription factor found in the nucleus in mice and in human cells (Du et al., 2013). We examined the partitioning of α1ACT into cytoplasmic and nuclear fractions (Fig. 2D). We observed that α1ACT11Q and α1ACT33Q are localized in both the cytoplasm and the nucleus. α1ACT70Q is also found in the nucleus, but its cytoplasmic presence is markedly lower compared with 11Q and 33Q variants (Fig. 2D), suggesting that the polyQ tract is important for the subcellular localization of α1ACT protein.

### PolyQ-dependent toxicity from α1ACT

**Fig. 3.**
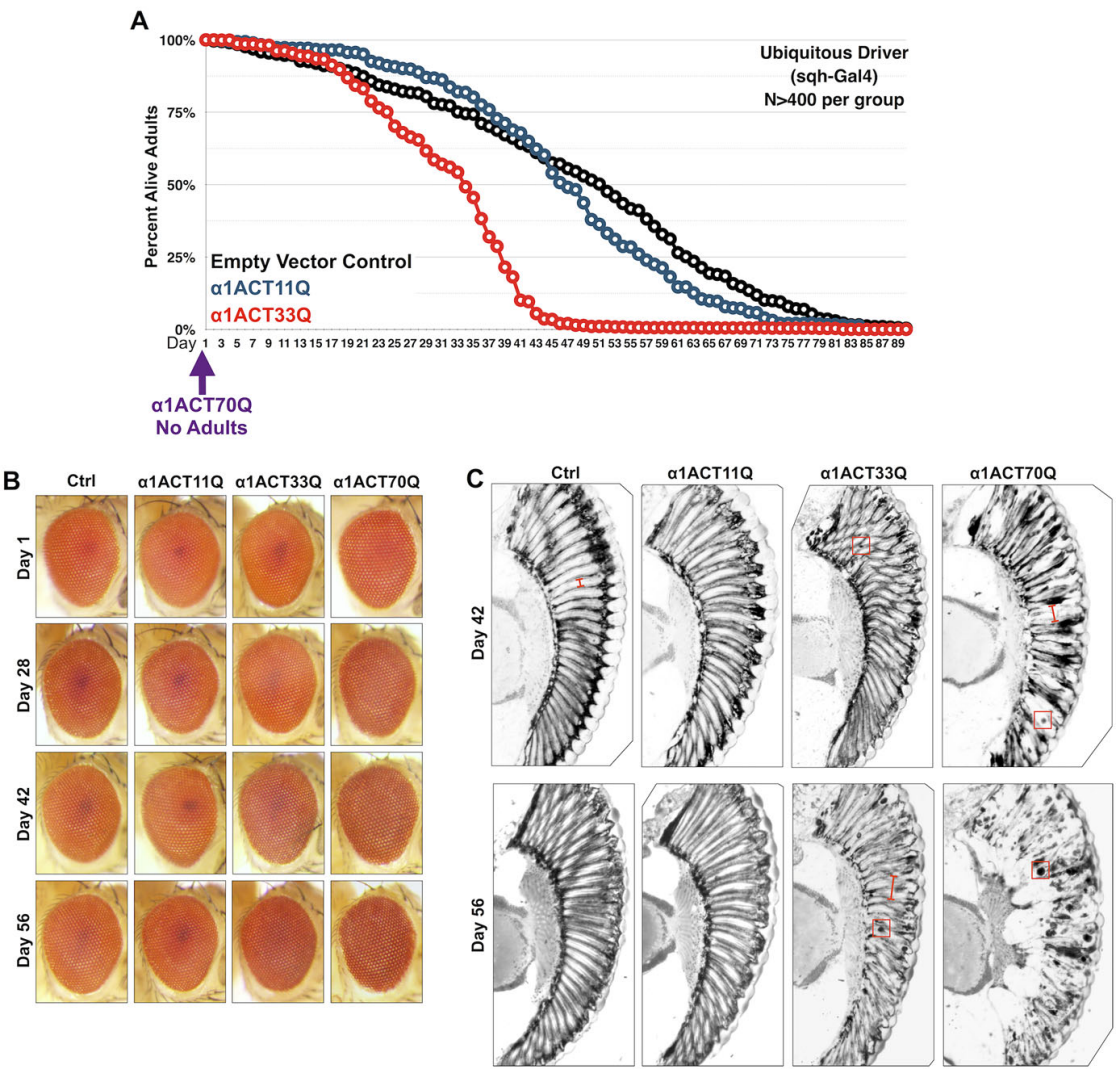
**PolyQ-dependent toxicity from α1ACT in *Drosophila*.** (A) Longevity of adult flies expressing the noted versions of α1ACT throughout the fly using the sqh-Gal4 driver. Flies were heterozygous for driver and α1ACT transgenes. (B,C) External eye photos (B) and histological sections (C) of α1ACT with 11Q, 33Q or 70Q driven by GMR-Gal4 in fly eyes. Ctrl indicates empty vector in the presence of GMR-Gal4. All flies were heterozygous for driver and α1ACT transgenes. Red boxes in sections indicate densely staining species; red bracketed lines in sections indicate ommatidial boundaries.

We tested the toxicity of α1ACT33Q when its expression was induced throughout the fly or selectively in a neuronal tissue, the fly eye. Expression of α1ACT33Q throughout development and in adults in a ubiquitous manner led to reduced longevity of adult flies. The driver used here was sqh-Gal4 (Franke et al., 2006, 2005; Todi et al., 2005, 2008). Flies expressing α1ACT33Q everywhere developed normally and eclosed successfully from their pupal cases, but were all dead by about 50 days (Fig. 3A). By comparison, flies expressing α1ACT11Q or ones that contain the host vector integrated into attP2 but that do not express any α1ACT protein lived up to nearly 90 days. Compared to these lines, expression of α1ACT70Q led to lethality at the pharate adult stage or during adult eclosure, and no adults came out successfully. These findings underscore the importance of polyQ length in toxicity from α1ACT when its CAG region is expanded beyond normal repeats.

When expressed selectively in fly eyes by the GMR-Gal4 driver, α1ACT33Q led to toxicity by eight weeks of age. Externally, there is some minor dyspigmentation that can be observed in 56-day-old flies, compared to control flies and ones expressing α1ACT11Q (Fig. 3B). Histologically, the signs of degeneration are clearer, and there is a loss of inter-ommatidial boundaries (Fig. 3C). Also present are densely-staining bodies (Fig. 3C). Aggregated structures can be seen as early as day 42 with α1ACT33Q. By comparison, expression of α1ACT70Q leads to a degenerative phenotype in retinal tissue at an earlier stage than α1ACT33Q and with more prominent, densely-staining structures. Compared to α1ACT with 33Q or 70Q, 11Q-expressing flies do not show histological anomalies; they are similar to control flies (Fig. 3B,C).

**Fig. 4.**
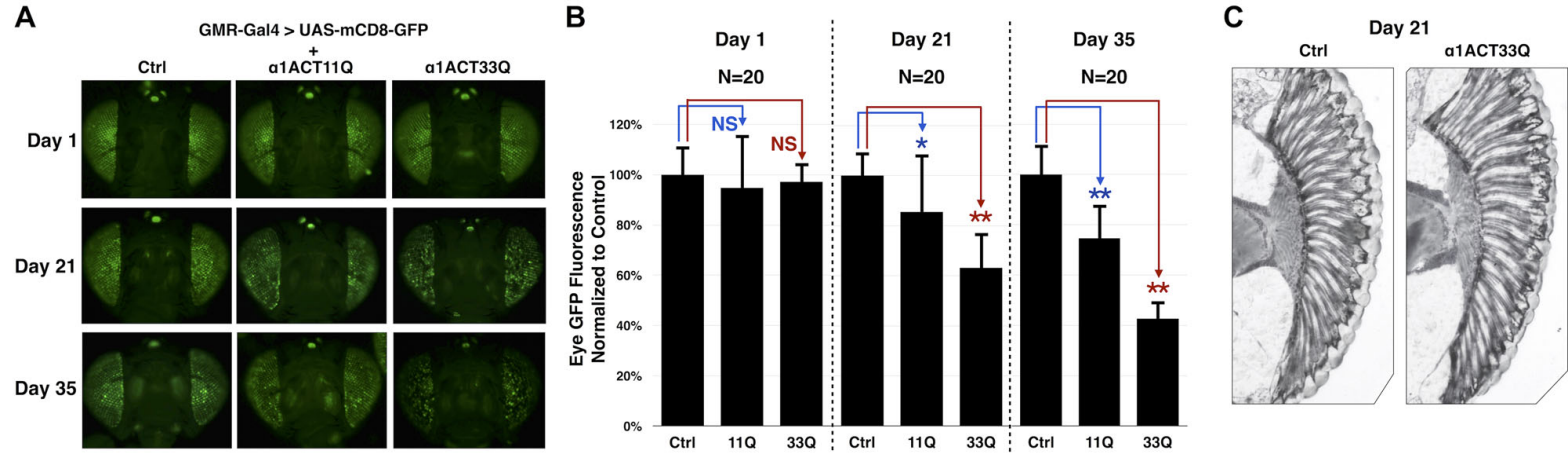
**Reduced GFP fluorescence in *Drosophila* eyes in the presence of α1ACT33Q.** (A) Representative photos of dissected fly heads expressing membrane-targeted CD8-GFP in the absence (Ctrl) or presence of α1ACT11Q or α1ACT33Q. Ctrl is an empty vector in the presence of GMR-Gal4 and CD8-GFP. Flies were heterozygous for all transgenes. (B) Quantification of data from A and other similar flies. Data shown as means with s.d. *P<0.05, **P<0.01, calculated using Student's *t*-tests. NS, non-statistically significant. (C) Histological sections of 21-day-old fly eyes without or with α1ACT33Q. Flies were heterozygous for all transgenes.

Lastly, we examined the time course of retinal degeneration using a more sensitive technique (Burr et al., 2014). In this method, fluorescence from membrane-targeted GFP serves as a surrogate of retinal integrity (Burr et al., 2014). GFP is expressed independently of α1ACT. The SCA6-related protein is expressed in its native, untagged form. As eyes degenerate, GFP fluorescence decreases, revealing mosaicism of the fluorescent compound fly eye. As summarized in Fig. 4, α1ACT33Q-expressing eyes appear normal, according to this technique, on day 1 after adults eclose from the pupal case. With age, fluorescence decreases significantly, as quantified in Fig. 4B. By 35 days, GFP fluorescence from α1ACT33Q is decreased to about 50% of non-α1ACT-expressing controls. By comparison, fluorescence from α1ACT70Q eyes is gone by 2 weeks of age (data not shown) (Tsou et al., 2015a).

As highlighted by Fig. 4C, the GFP-based method shows α1ACT33Q-dependent degeneration, as assayed by loss of fluorescence, on an earlier timescale than histology. At 21 days we do not observe clear histological marks of degeneration, but GFP fluorescence is already significantly lower (Fig. 4A,B). Thus, α1ACT33Q exhibits time-dependent toxicity in this neuronal tissue.

## DISCUSSION

We described an additional model of SCA6 in *Drosophila* that depends on the expression of the full-length α1ACT protein with a human disease range polyQ repeat of 33.

Compared with a hyper-expanded version with 70 polyQ, the current model (33Q) has a milder phenotype. Still, a clear, age-dependent phenotype is observable phenotypically (disruption of internal eye structures; shorter life span) and biochemically (SDS-resistant species that become more prominent over time) when the protein is expressed selectively in fly retinae or in all tissues. Fly eyes have been used frequently and successfully to understand the biology of disease of various toxic proteins and to screen for modifiers of these diseases (Bilen and Bonini, 2007; Blount et al., 2014; Bonini and Fortini, 2003; Casci and Pandey, 2015; Lanson et al., 2011; McGurk and Bonini, 2012; Park et al., 2013; Tsou et al., 2013, 2015b); our finding that α1ACT leads to eye degeneration in a time- and polyQ length-dependent manner is of importance to future work in SCA6.

When α1ACT is targeted to the eye, we also observe reduction in GFP fluorescence with the wild-type protein, something that we do not notice in histological sections. We have reported before that α1ACT11Q causes a reduction in GFP fluorescence (Tsou et al., 2015a). It is not uncommon for wild-type polyQ disease proteins to be toxic when expressed in *Drosophila* (Fernandez-Funez et al., 2000); this information may yield critical insight into the normal roles of these disease-related proteins that deserve future attention. However, the important point is that the polyQ-expanded versions are significantly more toxic than the wild-type counterparts, and that their toxicity increases significantly with longer polyQ stretches within an otherwise identical protein.

An intriguing point from the studies summarized in this report is the subcellular localization of α1ACT33Q compared with α1ACT70Q. According to cellular fractionation coupled with western blotting, the hyper-expanded version of α1ACT localizes less readily to the cytoplasm, whereas α1ACT11Q and α1ACT33Q show similar distribution to each other. Perhaps the longer polyQ repeat is recognized by specific nuclear transporters that lead to increased localization of this highly toxic protein into the nucleus, a site of toxicity for various polyQ proteins (Todi et al., 2007). Future work on potential factors that enhance the nuclear localization of over-expanded polyQ proteins into the nucleus may be of significance to polyQ diseases in general.

Collectively, these *Drosophila* lines for SCA6 provide a versatile and flexible system that should expedite our collective understanding of this disease and enable the generation of new therapies toward a cure for this neurodegenerative disorder.

## MATERIALS AND METHODS

### Antibodies

Anti-Tubulin (mouse monoclonal, 1:5000; Sigma-Aldrich, St Louis, MO, USA); anti-Lamin (mouse, monoclonal, ADL84.12; 1:200; Developmental Studies Hybridoma Bank at the University of Iowa, Iowa City, IA, USA); anti-α1A [rabbit polyclonal, 1:500; described previously (Du et al., 2013)]; ab32462 (rabbit polyclonal anti-α1A, 1:1000; Abcam, Cambridge, MA, USA); 1:5000 peroxidase-conjugated secondary antibodies (Jackson Immunoresearch, West Grove, PA, USA).

### *Drosophila* transgenics and stocks

Common-use stocks were procured from the Bloomington *Drosophila* Stock Center (Indiana University, IN, USA). Fly crosses were conducted at 25°C and ∼40-60% humidity in diurnal environments. To generate transgenic lines that express α1ACT33Q through the Gal4-UAS system (Brand and Perrimon, 1993), the DNA sequence covering exon 40 to the 3′ end of human *CACNA1A* was PCR-amplified using the primers 5′-ATGATCATGGAGTACTACCGGCAGA-3′ and 5′-TTAGCACCAATCATCGTCACTCTCG-3′, and inserted into the pWALIUM10-moe plasmid (DNA Resource Core at Harvard Medical School, MA, USA) by using EcoRI/BglII, as described previously (Tsou et al., 2015a). Constructs were injected by the Duke University Model System Injection Service into y[1], w[*]; +; attP2. For transgene verification, we extracted genomic DNA from different founder lines and PCR-amplified using primers white-end-F: 5′-TTCAATGATATCCAGTGCAGTAAAA-3′ and attP2-3L-R: 5′-CTCTTTGCAAGGCATTACATCTG-3′. All lines used here were on the same background. Table 1 details the genotypes of flies in each figure.

### Nuclear/cytoplasmic fractionation, lysis protocols and western blotting

Nuclear/cytoplasmic fractionation was done with the ReadyPrep Protein Extraction Kit (Bio-Rad, Hercules, CA, USA), as recommended by the manufacturer. In brief, 15 dissected adult fly heads per group were homogenized in 250 µl of cytoplasmic extraction buffer, incubated on ice for 2 min, spun quickly at 4°C, then supernatant was transferred and centrifuged at 1000×***g*** for 10 min at 4°C. Supernatant containing the cytoplasmic fraction was transferred into a fresh vial while the nuclear pellet was washed at least five times. The remaining nuclear pellet was then resuspended in 100 µl solubilization buffer. Protein loading buffer (6% SDS supplemented with final 50 mM dithiothreitol) was added to each sample, boiled for 5 min and loaded onto SDS-PAGE gels.

For western blotting of dissected adult fly heads, 15 heads per group were homogenized in boiling SDS lysis buffer (50 mM Tris pH 6.8, 2% SDS, 10% glycerol and 100 mM dithiothreitol). This buffer was chosen based on earlier work, where we found that it provided the highest quantity of SDS-soluble and SDS-resistant species of polyQ peptides (Tsou et al., 2013). Heads were mechanically homogenized with a pestle, sonicated for fifteen seconds, boiled for 10 min, centrifuged at 13,300×***g*** at room temperature for 10 min, and supernatant was loaded onto SDS-PAGE gels. Western blots were developed using a CCD-equipped VersaDoc 5000MP system (Bio-Rad).

### Longevity assay

At least 400 adults were collected and aged in conventional fly media. Adults were transferred to fresh vials every 2-3 days, until the day when all adults died.

### Fluorescence measurements

All fluorescence images were taken with an Olympus BX53 microscope and CellSens software (Olympus, Waltham, MA, USA). The same objective (10×) and camera settings (ISO 200, 500 ms capture time for each sample) were used for all images. Image capture and quantification (ImageJ, NIH) were conducted by separate investigators. Control and experimental flies were all imaged on the same day. ImageJ's freehand tool was used to outline and to quantify fluorescence readings from each fly eye.

### Histology

Adult flies whose proboscises and wings were removed were fixed in 2% glutaraldehyde/2% paraformaldehyde in Tris-buffered saline overnight at 4°C. Fixed flies were later dehydrated in a series of 30, 50, 75 and 100% ethanol and propylene oxide, embedded in Poly/Bed812 (Polysciences, Warrington, PA, USA), sectioned at 5 µm and then stained with Toluidine Blue.

### Quantitative RT-PCR

Total RNA from dissected adult heads was extracted by using TRIzol reagent (Life Technologies, Waltham, MA, USA). Extracted RNA was treated with TURBO DNAse (Ambion, Waltham, MA, USA) to remove contaminating DNA. Reverse transcription was performed with the High-Capacity cDNA Reverse Transcription Kit (ABI, New York, NY, USA). Messenger RNA levels were quantified by using the StepOnePlus Real-Time PCR System with Fast SYBR Green Master Mix (ABI). rp49 was used as internal control. Primers: α1ACT-F: 5′-CTAACTCTCAGTCCGTGGAGATG-3′; α1ACT-R: 5′-GTCTGAGATGGTACTGAGGTTATTCC-3′; rp49-F: 5′-AGATCGTGAAGAAGCGCACCAAG-3′; rp49-R: 5′-CACCAGGAACTTCTTGAATCCGG-3′.
